# Macula Densa Alleviates Shiga Toxin-Induced Acute Kidney Injury via CCN1-Mediated Renal Tubular Repair

**DOI:** 10.3390/toxins17090470

**Published:** 2025-09-21

**Authors:** Hongzhi Wan, Yuhui Wang, Jiahui Chen, Hongqi Liu, Jiamei Li, Qisheng Su, Hui Peng, Xiaotao Duan, Bo Wang

**Affiliations:** 1 Academy of Military Medical Sciences, Beijing 100850, China; 2 Military Medical Sciences Academy, Tianjin 300050, China; 3 School of Pharmacy, Shenyang Pharmaceutical University, Shenyang 110016, China; wangyh16308@163.com (Y.W.); hazel912124@163.com (J.C.); 4 Medical School of Chinese PLA, Beijing 100850, China; hotpot7777@126.com; 5 Department of Stomatology, The First Medical Center of Chinese PLA General Hospital, Beijing 100850, China; 6 School of Pharmacy, Qingdao University, Qingdao 266071, China; 7 Department of Clinical Laboratory, First Affiliated Hospital Guangxi Medical University, Nanning 530021, China; suqisheng@gxmu.edu.cn

**Keywords:** Shiga toxin, macula densa, human kidney organoids, CCN1, Renal tubular repair

## Abstract

Shiga toxins (Stx), produced by Shiga toxin-producing *Escherichia coli*, preferentially attack renal tissue and frequently induce acute kidney injury (AKI) and renal failure. To prevent irreversible damage, the injured renal tissue, particularly renal tubular epithelium, mounts a remodeling and regeneration response to repair itself. However, how such intrinsic renal repair processes are initiated and coordinated in infected renal tubular regions remains elusive. Herein, we reported that macula densa apparatus, in addition to its conventional role as a salt sensor in nephron, can function as an endogenous sensor for exogenous toxins (e.g., Stx). We demonstrated that macula densa cells orchestrate a rapid repair niche by initiating transcriptional activation of repair and regeneration factors in both Stx-injured murine models and human kidney organoids. Mechanistically, we showed that in response to Stx exposure, macula densa cells release a specific repair factor CCN1, which effectively promotes the regeneration of toxin-injured renal tubular epithelium and facilitates renal tubular repair through integrin-mediated signaling pathways. Moreover, we demonstrated that treatment with recombinant CCN1 can greatly ameliorate the structural damage and significantly restore the proximal tubular reabsorption capacity in Stx-infected kidney organoids. Our finding highlights a novel role of macula densa apparatus in toxin-induced renal injury, and paves a new avenue for treatment of AKI-associated renal diseases.

## 1. Introduction

Shiga toxin-producing *Escherichia coli* (STEC), a pathogenic bacterium widely distributed in the environment, poses a significant threat to human health through food- or water-borne transmission [1]. STEC infections can progress to hemolytic uremic syndrome (HUS), which is the leading cause of renal failure in infants and young children [2]. Presently no therapeutics effective against STEC or *Shigella* infection exist, and antibiotics are contraindicated in the clinical management of STEC infections [3]. Shiga toxins (i.e., Stx1 and Stx2) are the principal virulence factors of STEC. They specifically recognize the receptor globotrihexosylceramide (Gb3), which extensively expresses on the renal glomerular endothelial cells and renal tubular epithelium in humans [4,5]. The Gb3-directed Stx renal exposure typically results in renal tubular epithelium damage, renal tubular obstruction, and eventually acute kidney injury (AKI) [6].

Of note, in case of irreversible damage, the injured renal tissues spontaneously initiate activation of endogenous self-repair process at the early stage of AKI [7,8,9,10,11]. More interestingly, the repair process, occurring frequently on renal tubular epithelium, is predominantly mediated by intrinsic renal cells (e.g., the surviving renal tubular epithelial cells, RTECs), instead of through differentiation of intratubular epithelial progenitor cells [9]. Genetic lineage analysis further confirmed this observation showing that renal tubular cells, rather than extra tubular cells, are the principal endogenous drivers of renal repair after acute tubular damage [8]. The renal tubular apparatus comprises multiple distinct segments—including the proximal tubule, descending thin limb of Henle, ascending thin limb of Henle, macula densa, connecting tubule, and others—each populated by heterogeneous epithelial subtypes with unique molecular profiles [12,13]. How these various sub-organ structures and cell types sense damage signals and how they coordinate with each other to orchestrate an efficient repair niche remain largely unknown.

The macula densa (MD) apparatus comprises a subset of renal tubular epithelial cells that is capable of modulating renal tubular reabsorption and maintaining hemodynamic and electrolyte balance in kidney. The MD cell cluster anatomically resides at the distal convoluted tubule adjacent to the glomerular vascular pole [14]. As a core component within the renin–angiotensin–aldosterone system (RAAS), it engages in sensing endogenous salt concentration and releasing PTGS2-derived prostaglandin E2 (PGE2) to regulate renin secretion from juxtaglomerular cells [15]. Herein, we found that in addition to their classical role as salt sensor, macula densa cells possess an intrinsic ability to sense and respond to exogenous toxins. We demonstrated that macula densa cells orchestrate rapid tissue repair by initiating transcriptional activation of repair and regeneration factors in both Stx-injured murine models and human kidney organoids. Specifically, we showed that upon Stx exposure, macula densa cells immediately release a specific renal repair factor CCN1, which effectively promotes the regeneration of toxin-injured renal tubular epithelium and facilitates renal tubular repair through integrin-mediated signaling pathways. Moreover, we demonstrated that treatment with recombinant CCN1 can significantly alleviate the symptoms and severity of renal damage in Stx2-induced AKI.

## 2. Results

### 2.1. Macula Densa Apparatus Displays Transcriptional Signatures Associated with Renal Repair and Remodeling in Stx-Induced Renal Injury

**Figure 1:**
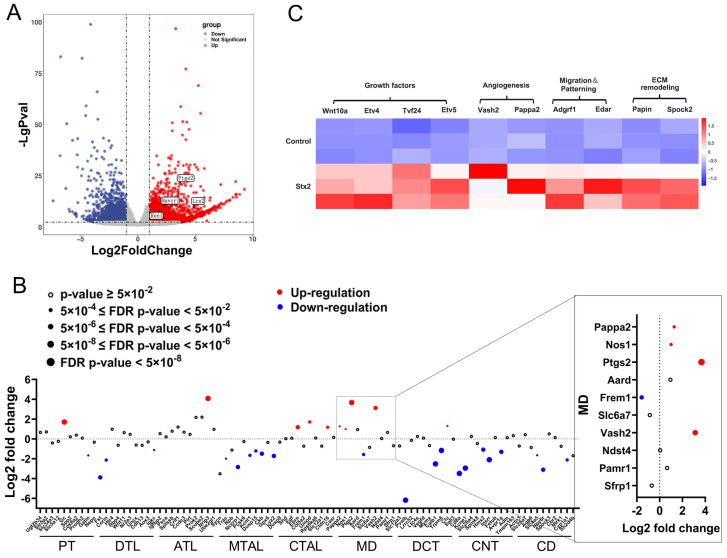
Genome-wide transcriptional profiling reveals macula densa involvement in renal repair and remodeling in Stx-induced renal injury. (**A**) Volcano plot showing differentially expressed genes (DEGs) in Stx2-induced nephrotoxicity (bulk RNA-seq). RNA reads were aligned to the mouse reference genome (GRCm39, Ensembl), with genes exhibiting a fold change > 2 (marked by vertical dashed lines), *p*-value < 0.05 (indicated by the horizontal dashed line) considered significant. Up-regulated genes are highlighted in red, downregulated genes in blue, and non-significant genes in gray. (**B**) Dot plot of DEGs 72 h after Stx2 administration compared with the control. Dot size reflects significance (based on the *p*-value); with open circle indicating *p* > 0.05. Red and blue denote upregulated and downregulated genes, respectively. Abbreviations: PT, proximal tubule; DTL, descending thin limb of Henle; ATL, ascending thin limb of Henle; MTAL, medullary ascending limb of Henle; CTAL, cortical ascending thin limb of Henle; MD, macula densa; DCT, distal convoluted tubule; CNT, connecting tubule; CD, collecting duct. (**C**) Heat map of top enriched MD-specific gene expression across samples. Expression levels (FPKM, Fragments Per Kilobase of transcript per Million mapped reads) are represented by a color gradient, with high expression in red and low expression in blue. Columns correspond to genes, and rows represent individual samples.

We constructed a murine acute kidney infection model by intraperitoneal injection of Stx2 in C57BL6 mouse at LD50 dose (100 ng/kg). C57BL6 mice were sacrificed 72 h after a single *i.p.* injection of Stx2 solution or equal volume of PBS (control), and their renal cortex tissues were harvested for subsequent analysis. For genome-wide transcriptional profiling, we utilized the mouse reference genome (GRCm39) from the Ensembl database for functional annotation of detected transcripts. Candidate differentially expressed genes (DEGs) were filtered using strict criteria: fold change > 2, *p*-value < 0.05, and adjusted *p*-value (padj) < 0.05. This analysis identified a total of 3577 DEGs in renal tissues from Stx2-infected mice compared to those from control mice (Figure S1A). The transcript levels of *Havcr1* (*Kim-1*) and *Lcn2* (*Ngal*), both well-validated biomarkers of AKI, were found markedly increased in Stx2-treated group, demonstrating the successful establishment of the injury model (Figure 1A). KEGG and GO analysis reveals enrichment of previously described pathways associated with Stx infection, including cytokine-cytokine receptor interaction, chemokine signaling, extracellular matrix organization, as well as cell–matrix adhesion (Figure S1B,C). Considering renal tubules are the preferential target of Stx, we attempted to take a closer look at the transcriptional levels of genes related to renal tubules and RAAS. We examined the bulk transcript data of renal tubules after grouping according to the sub-segments of renal tubules (Figure 1B). The transcripts of representative genes for each renal tubule segment were extracted and analyzed. Of note, we observed a significant up-regulation of *Ptgs2*, a macula densa gene responsible for PGE2 synthesis, in Stx2 treated group, which preceded up-regulation of *Ren1* and renin release in juxtaglomerular cells. This observation indicates that Stx challenge activated the adaptive regulation of macula densa-driven signaling axis (i.e., RAAS) in infected renal tissues. More interestingly, we found that macula densa segment in Stx-treated group displays unique transcriptional signatures associated with renal repair and remodeling (Figure 1C). For instance, we observed significant up-regulation of macula densa genes functionally involved in angiogenesis (e.g., *Pappa2*, *Vash2*), cell migration and patterning (e.g., *Adgrf1*, *Edar*), transcription and growth factors (e.g., *Wnt10a*, *Etv4*, *Tcf24*, *Etv5*), and extracellular matrix remodeling (e.g., *Papln*, *Spock2*). Given the limited number of samples available, we conducted further validation of several key genes that showed differential expression in the sequencing analysis using RT-qPCR. The results of these validations were consistent with the sequencing data, reinforcing the reliability of our findings (Figure S1D). These findings collectively suggest that macula densa cells response actively to Stx stimuli and may play a protective role in Stx2-induced AKI.

### 2.2. Stx Infection Induces Transcriptional Activation of Macula Densa-Derived Repair Factors in Human Renal Organoids

**Figure 2:**
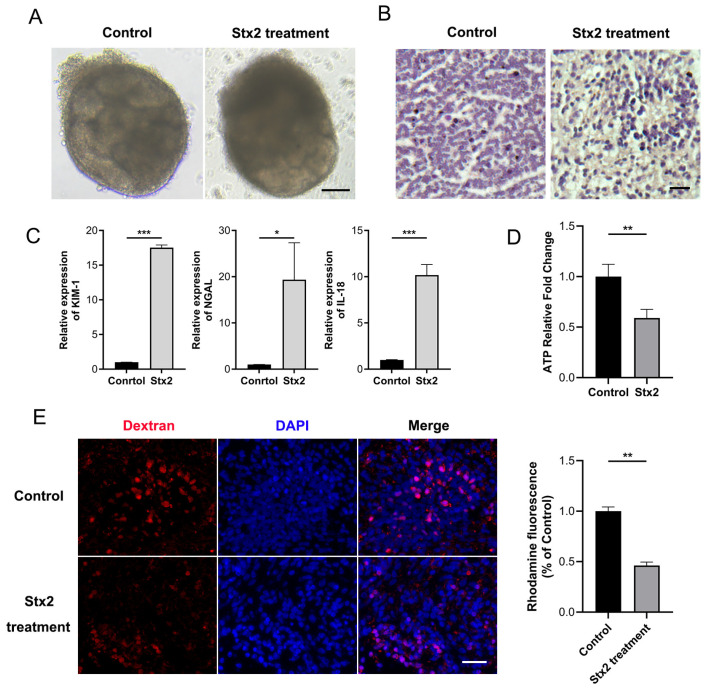
Establishment of an Stx2-induced human kidney organoid injury model. (**A**) Microscopic images of human kidney organoids treated with Stx2 or PBS (control) for 108 h. Scale bar, 50 μm. (**B**) Human kidney organoid sections were stained with H&E. Scale bar, 20 µm. (**C**) Relative mRNA expression levels of acute kidney injury markers in Stx2 treated human kidney organoids, quantified by RT-qPCR. * *p* < 0.05, *** *p* < 0.001. (**D**) Intracellular ATP levels in organoids, measured using the CellTiter-Glo 3D Cell Viability Assay. Values represent mean ± standard deviation; *n* = 3 with technical triplicates. *n* = 3 with technical triplicates. ** *p* < 0.01. (**E**) Uptake of 10 kDa dextran-rhodamine (red) in human kidney organoids treated with Stx2 or PBS. Nuclei were counterstained with DAPI (blue). Quantitative Analysis of Rhodamine Fluorescence Intensity. Scale bar, 20 µm. All experiments had *n* = 3 independent biological replicates per group. ** *p* < 0.01.

Considering the interspecies differences, we attempted to establish the Stx-induced injury model using human renal organoids. We generated human renal organoids from human induced pluripotent stem cells (hiPSCs) as described [16]. As expected, complex tubular structures became visible by wide-field microscopy at day12 (Figure S1E). Whole organoid immunofluorescence showed the presence of segmenting nephron structures including glomerulus, proximal tubule, and distal tubule, etc. The fluorescent ATP assay and dextran uptake assay confirmed that our developed human kidney organoids are mature and functional, and thus feasible for the following Stx tests. Clinically, HUS typically manifests approximately 5 days after disease onset. We thus selected a 4.5-day time window to capture the early pathogenic processes that precede the overt clinical symptoms of HUS. Next, we added 10 ng/mL of Stx2 to the culture medium of kidney organoids that had been cultured for 12 days and collected sample 108 h post treatment. At this time point, we found that the kidney organoids exposure to Stx2 displayed a clearly damaged morphology with disrupted tubular structures and smeared segmentation (Figure 2A,B). Additionally, we observed significantly elevated levels of AKI biomarkers in Stx2 treated group (Figure 2C). The acute increase in individual injury markers (e.g., *KIM-1* for proximal tubules [17], *IL-18* for distal tubules, connecting tubule, and collecting ducts [18,19], *NGAL* for Lis of Henle [20]) suggests an extensive injury across multiple segments of the renal tubules in human kidney organoids. ATP measurement (by CellTiter-Glo 3D cell viability assay) revealed a significantly lower ATP level and organoid viability in Stx-2 treated group compared with control samples (Figure 2D). Moreover, we observed a severe impairment of the tubular reabsorption in Stx-2 treated organoids, as assessed by fluorescent dextran uptake assay (10 kDa rhodamine-labeled dextran) (Figure 2E). These observations generally complied with the characteristics of AKI in humans, demonstrating the establishment of a Stx2-indcued organoid injury model.

**Figure 3:**
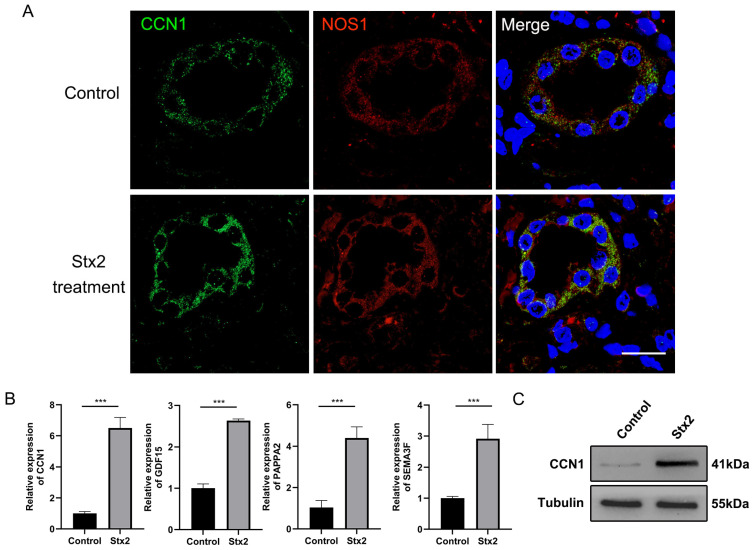
Stx2 infection induces the upregulation of the macula densa-specific factor CCN1 in human kidney organoids. (**A**) Immunofluorescence double staining for NOS1 (green) and CCN1 (red), showing expression of both proteins in the same cells. Nuclei were counterstained with DAPI (blue). Scale bar, 20 µm. (**B**) Relative mRNA expression levels of MD-specific factors in Stx2 treated human kidney organoids following Stx2 treatment, quantified by RT-qPCR. *** *p* < 0.001. (**C**) Western blot analysis of CCN1 protein expression in human kidney organoids treated with Stx2 or PBS. Tubulin was used as a loading control. All experiments had *n* = 3 independent biological replicates per group.

We labeled macula densa cells with NOS1, a well-established marker mainly expressed in the macula densa, by immunofluorescence in human renal organoids (Figure 3A). We observed that while Stx2 treatment did not induced apparent apoptosis or necrosis of macula densa cells as reflected by the fluorescence intensity, it profoundly upregulated the transcript levels of a plethora of macula densa-specific genes which are involved in tissue regeneration and angiogenesis (e.g., *PAPPA2*, *CCN1*, *SEMA3F* and *GDF15* as shown in Figure 3B). Of them *CCN1* showed the highest level of upregulation (∼6-fold on average, *n* = 6) in response to Stx2. We further confirmed the Stx2-indcued upregulation of CCN1 also occurs at protein level (Figure 3C). Interestingly, we noticed that NOS1-positive cells are constantly co-stained with CCN1 on organoid tissue slices (Figure 3A), in agreement with the fact that CCN1 is a matricellular protein specifically produced and secreted by macula densa cells [21]. Recently emerging evidences have suggested that CCN1 may play an important role in tissue remodeling and repair associated with several injuries [22,23]. We therefore envision that macula densa cells can orchestrate an efficient repair and regeneration niche by rapidly expressing repair factors such as CCN1 to counteract Stx2-induced AKI.

### 2.3. Macula Densa-Derived Niche Factor CCN1 Promotes the Proliferation and Regeneration of Stx-Injured RTECs

**Figure 4:**
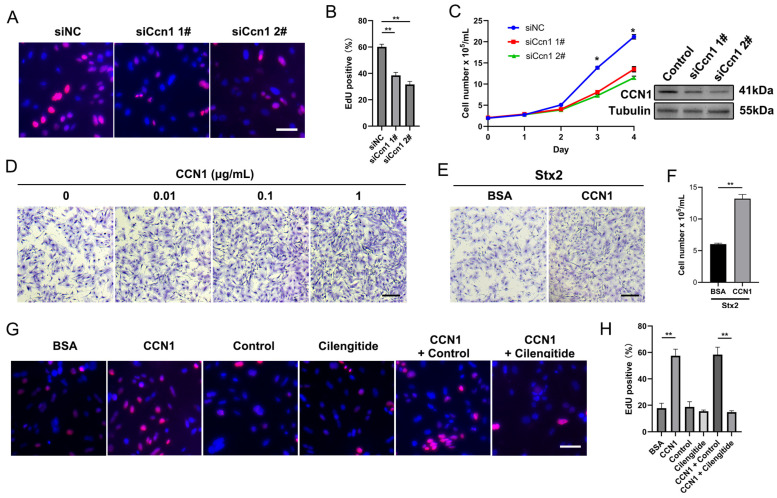
CCN1 promotes the proliferation and regeneration of Stx-injured RTECs. (**A**) Representative images of EdU incorporation assay in RTECs. Nuclei are stained blue (DAPI), and proliferating cells (EdU-positive) are labeled red. (**B**) Quantification of EdU-positive cells expressed as a percentage of total nuclei. ** *p* < 0.01, Statistical significance was determined using one-way ANOVA followed by Tukey’s HSD post hoc test. (**C**) Western blot confirming CCN1 knockdown in RTECs transfected with CCN1-targeting siRNA (siCcn1) versus non-targeting control (siNC). Growth curve shows effects of siCcn1. * *p* < 0.05, two-sided Student’s *t*-test. (**D**) Crystal violet staining of RTECs cultured for 24 h with recombinant CCN1 protein at indicated concentrations. Scale bar, 20 µm. (**E**) Crystal violet staining of RTECs pretreated with Stx2 (100 ng/mL, 24 h) followed by 24 h culture with CCN1 (1 µg/mL) or BSA control. Scale bar, 20 µm. (**F**) The number of RTECs was assessed by trypan blue staining. ** *p* < 0.01, two-sided Student’s *t*-test. (**G**) EdU incorporation assay in RTECs treated with BSA (1 µg/mL), CCN1 (1 µg/mL), cilengitide (0.5 µM), or PBS. Nuclei (blue) and proliferating cells (red) are shown. Scale bar, 20 µm. (**H**) Quantification of EdU-positive cells under treatments described in (**G**). ** *p* < 0.01, Statistical significance was determined using one-way ANOVA followed by Tukey’s HSD post hoc test.

To verify the effect of macula densa-derived CCN1 in renal tubular regeneration, we isolated primary mouse RTECs via collagenase-based digestion and validated their identity through bright-field imaging and immunofluorescence as described before [24]. We confirmed that a typical polygonal cobblestone-like morphology and a high purity of RTECs were achieved in our preparation (EPCAM+ rate > 90%) (Figure S1F,G). Next, we performed genetic knockdown of endogenous Ccn1 in RTECs via small interfering RNAs (siRNAs, targeting non-overlapping parts of Ccn1 mRNA), and measured the cell proliferation with EdU incorporation assay. We observed that CCN1 knockdown significantly suppressed the proliferation rate of RTECs (Figure 4A,B). Trypan blue-based cell counts confirmed this observation (Figure 4C). In turn, we found that the exogenous addition of recombinant CCN1 protein to mouse RTECs cultures (at final concentrations of 0.01~1.0 μg/mL) profoundly enhanced the cell proliferation rate in a dose-dependent manner (Figure 4D). More importantly, in the context of Stx2 exposure, the addition of recombinant CCN1 largely restore the impairment of RTECs’ survival and proliferation induced by Stx2 toxin (Figure 4E,F). Integrin αvβ5 is the receptor of CCN1 on renal tubular mediating renal tissue differentiation and regeneration, and Cilengitide is reported to be able to specifically block the binding of CCN1 to its receptor Integrin αvβ3/αvβ5 [22,25]. In our settings, we found that Cilengitide treatment nearly abolished the pro-proliferation effects of CCN1 on RTECs (Figure 4G,H). These data together suggested that macula densa-derived niche factor CCN1 promotes the proliferation and regeneration of Stx-injured renal tubular epithelial cells through Integrin αvβ5-mediated signaling.

### 2.4. CCN1 Alleviates Stx2-Induced Renal Injury in Human Kidney Organoids

**Figure 5:**
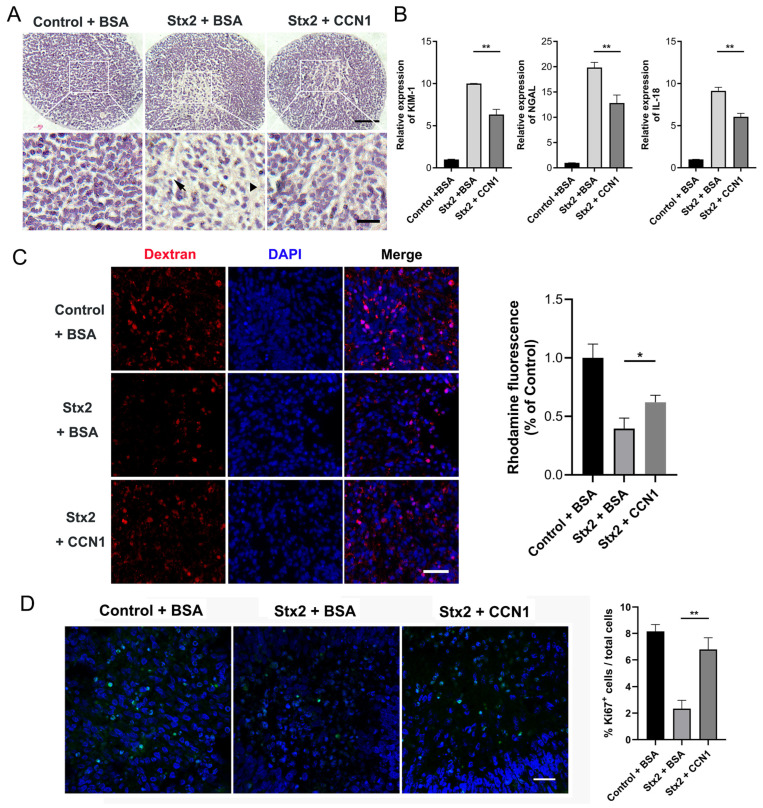
CCN1 ameliorates the structural damage and restores the tubular reabsorption capacity in Stx-infected kidney organoids. (**A**) H&E-stained sections of human kidney organoids under different treatments: control + BSA, Stx2 + BSA, and Stx2 + CCN1. Arrows mark tubular cell shedding, and triangles indicate regions with apparent tubular dilatation Scale bar, 50 µm, 15 µm. (**B**) Relative mRNA expression levels of AKI factors in Stx2-treated human kidney organoids quantified by RT-qPCR. ** *p* < 0.01. (**C**) Uptake of 10 kDa dextran-rhodamine (red) in human kidney organoids. Nuclei were counterstained with DAPI (blue). The organoids were divided into three groups: control + BSA, Stx2 + BSA, and Stx2 + CCN1. Quantitative Analysis of Rhodamine Fluorescence Intensity. Scale bar, 30 µm. * *p* < 0.05 (**D**) Ki67 immunofluorescence staining in human kidney organoid paraffin sections. Ki67-positive cells (green fluorescence) indicate proliferating cells. Nuclei were counterstained with DAPI (blue). Quantification of Ki67-positive cells in different treatment groups. Scale bar, 20 µm. ** *p* < 0.01. All experiments had *n* = 3 independent biological replicates per group.

To further substantiate the protective effect of CCN1 in Stx2-induced renal injury, we administered recombinant CCN1 to human kidney organoids following Stx2-induced injury. Specifically, we infected human renal organoids with Stx2 as aforementioned. After 24 h either recombinant CCN1 (at final concentration of 1.0 μg/mL) or placebo was added to the culture medium of kidney organoids. Organoid samples were further cultured until 108 h after the onset of Stx2 treatment. Then we performed H&E staining on the infected organoid sections. We found that while Stx2 induced apparent tubular dilatation (Figure 5A, indicated with triangles) and epithelial cell shedding (Figure 5A, indicated with arrows), the Stx-damaged tubular structures were largely restored in the CCN1-treated groups (Figure 5A). We examined the expression levels of established biomarkers associated with AKI. *KIM-1*, *NGAL* and *IL-18* were significantly increased exposed to Stx2 compared to intoxicated organoids treated with CCN1 (Figure 5B). Moreover, we implemented the fluorescent dextran uptake assay to investigate the reabsorption of renal tubular. We found that compared to the control + BSA group, human kidney organoids treated with Stx2 + BSA exhibit a marked reduction in the Rhodamine-positive fraction, indicating impairment of organoids reabsorption capacity. Administration of recombinant CCN1 partially reversed this toxic effect, yielding higher fluorescence intensity that signifies the restoration of organoids reabsorptive function (Figure 5C). Ki67 immunofluorescence staining showed that the percentage of Ki67-positive cells in Stx2-injured renal organoids was significantly lower than that in the control + BSA group. In contrast, treatment with recombinant CCN1 notably elevated the percentage of Ki67-positive cells, indicating that CCN1 can promote cell proliferation and regeneration in renal organoids after Stx2-induced injury (Figure 5D). These findings together suggested that CCN1 effectively alleviates Stx2-induced renal injury in human organoids.

## 3. Discussion

Renal tubular epithelial cells play pivotal roles in the maintenance of the normal structure and function of the healthy renal tubular tissue, and also in the development of renal tubular injury in infection diseases (such as STEC or Shigella infections). However, our knowledge of the mechanisms that constantly maintain and regenerate the renal tubular epithelium after toxin-induced injury has been limited. It is partly due to the lack of infection models that can mimic the complex injury and repair responses in human kidney. We constructed a kidney injury model using human renal organoids derived from hiPSCs [16]. This model well recapitulates the clinical features of toxin-induced acute renal injury including shedding of tubular cells, elevated injury biomarkers, impaired cellular metabolism, and disrupted tubular reabsorption, etc. In this manuscript, we successfully applied this injury model to investigate the renal repair process in response to Stx infection. Previous studies have established that renal tubular repair after injury primarily relies on endogenous epithelial cells rather than cells derived from the bone marrow-derived or renal interstitial cell-derived epithelial progenitor cell population [8,9]. However, a comprehensive understanding of the underlying mechanisms, particularly the interaction of multiple renal apparatuses, remains elusive.

As a specialized structure within the juxtaglomerular apparatus (JGA), macula densa apparatus has both high secretary activity and protein synthesis activity [26]. Notably, PTGS2 and NOS1 are predominantly expressed in the macula densa, though they are also occasionally detectable in other renal apparatuses like the cortical thick ascending limb and glomerular podocytes—especially under injury conditions. Under physiological conditions, macula densa apparatus is primarily responsible for the regulation of renin secretion from juxtaglomerular cells by releasing PTGS2-derived PGE2. Our study shows that the macula densa rapidly responses to Stx and regulates tissue remodeling and regeneration in toxin-induced acute kidney injury. This effect is independent of its conventional role in salt sensing and regulation of renin release. The unique repair capacity of macula densa has also been observed in a Adriamycin-induced chronic kidney injury model recently reported by Gyarmati et al.

The matricellular protein CCN1 has recently emerged as an important multifunctional regulator of repair and regeneration process. For instance, it promotes wound healing in skin by enhancing cell proliferation and migration [23]. It mediates the repair response in cholestatic liver injury by facilitating cholangiocyte proliferation and ductular reactions [22]. In our study, we identified that CCN1 is acutely produced and secreted by macula densa in response to toxin stimuli in both Stx-infected mouse model and human renal organoids. It is thus emerging as a key injury response molecule that coordinates multiple aspects of renal tissue repair. We further demonstrated that recombinant CCN1 protein can effectively promote proliferation and regeneration of Stx-injured RTECs, and moreover, greatly attenuate the renal tubular injury and restore the tubular re-absorption capacity in Stx-infected renal organoids. The protective effect of CCN1 towards renal tubular epithelium is mechanistically linked to integrin signaling pathway, as it can be completely blocked by Cilengitide (an inhibitor of CCN1 receptor integrin αvβ5).

It should be noted, however, that the renal organoid model used in our study has several limitations, for example, it lacks a functional vascular network and resident immune cells, which are critical components of the human kidney microenvironment and may influence the injury progression and repair dynamics in vivo. In addition, Stx2-induced tissue damage encompasses diverse forms of cell death, including apoptosis [27], necrosis [28,29], ferroptosis [30,31], pyroptosis [32,33,34], and necroptosis [35], which contribute to the multi-faceted pathological changes observed in our organoid model. Therefore, a comprehensive investigation to delineate the complexity of cell death in Stx-induced acute renal injury model is warranted in our future study.

## 4. Conclusions

Overall, our work identifies a novel role of macula densa as an endogenous sensor of toxins (e.g., Stx) which creates a repair niche and mediates an efficient repair process to counteract renal injury in early stage of Stx infection. Moreover, our work highlights the therapeutic potential of macula densa-specific repair factor CCN1 which accelerates the regeneration of renal tubular epithelium and attenuate acute injury in Stx2-induced AKI. There is at present only supportive care and no specific treatment for established AKI in infection contexts (e.g., STECs). Promoting macula densa-mediated endogenous tissue repair may represent a novel therapeutic approach for toxin-associated renal diseases.

## 5. Materials and Methods

### 5.1. Experimental Animal Group Design and Sample Collection

Male wild-type C57BL/6 mice (Vital River, Beijing, China) were housed in a controlled environment maintained at 22–24 °C with a 12 h light/dark cycle and 50–60% humidity. The mice were fed a standardized pelleted diet and provided with ad libitum access to water. The mice were randomly divided into two experimental groups (*n* = 3 per group): the Stx2-treated group received an intraperitoneal injection of 100 ng/kg Shiga toxin 2 (Stx2; Mighty Biotechnology Company Limited, Nanjing, China), while the control group received an equivalent volume of sterile saline. The Stx2 dosage was selected based on previously reported LD50 values for rodents [36,37]. At 72 h post-injection, kidney samples were harvested for subsequent analysis.

### 5.2. Human Kidney Organoids Culture

Human kidney organoids were generated from induced pluripotent stem cells (hiPSCs) using a modified suspension culture protocol [16]. On Day 0, hiPSCs at 60–70% confluence were washed twice with DPBS and dissociated into single cells using Accutase (Innovative Cell Technologies, San Diego, CA, USA) for 5 min at 37 °C. The cells were resuspended in BPEL medium [38], modified with 0.1 × ITS-X [39], supplemented with 8 μM CHIR99021, 3.3 μM Y27632, and 0.1 mM β-mercaptoethanol, then plated in ultra-low attachment 96-well plates (Corning, New York, NY, USA) to promote embryoid body (EB) formation. On Day 2, aggregated EBs were transferred to ultra-low attachment 6-well plates (Corning) and half of the medium was replaced with fresh BPEL medium containing 8 μM CHIR99021. On Day 3, EBs were transitioned to Stage II medium (DMEM supplemented with 15% KOSR [Gibco], 1% non-essential amino acids, 1% penicillin/streptomycin, 1% HEPES, 1% GlutaMAX, 0.05% polyvinyl alcohol, and 2.5 μg/mL Plasmocin). The medium was refreshed every 48 h until organoids reached maturity.

### 5.3. Quantitative Reverse Transcription PCR (qRT-PCR Assay)

Total RNA was extracted from cells or tissues using the Total RNA Extraction Kit (TIANGEN, Beijing, China) and quantified. Reverse transcription was performed using the PrimeScript RT Reagent Kit (TaKaRa, Kusatsu, Japan). qRT-PCR was conducted using SYBR Green Master Mix (Thermo Fisher Scientific, Waltham, MA, USA). Reactions were performed in triplicate via SYBR Green-based qRT-PCR. Cycling conditions were 10 min at 95 °C for activation/denaturation, followed by 40 cycles of 15 s at 95 °C (denaturation) and 1 min at 60 °C (annealing/extension). Post-amplification, a melting curve analysis (60–95 °C at 0.5 °C/5 s) verified amplicon specificity. Gene expression was normalized to glyceraldehyde 3-phosphate dehydrogenase (GAPDH) and analyzed via the ΔΔCt method. Primer sequences are provided in Table 1.

### 5.4. Western Blot Analysis

Cells or human kidney organoids were lysed in RIPA buffer (P0013B, Beyotime, Shanghai, China) for protein extraction. Protein concentrations were determined using a BCA Protein Assay Kit (23225, Thermo Fisher Scientific). Following heat denaturation, equal amounts of protein were separated by SDS-PAGE and transferred to polyvinylidene fluoride (PVDF) membranes (PVH00010, Millipore, Burlington, MA, USA). The membranes were blocked with 5% skim milk in TBST for 1 h at room temperature, followed by incubation with primary antibodies against Tubulin (T5168, Sigma-Aldrich, St. Louis, MO, USA; 1:1000 dilution) and CCN1 (A1111, ABclonal, Wuhan, Hubei, China; 1:1000 dilution) overnight at 4 °C. After three washes with TBST, the membranes were incubated with HRP-conjugated secondary antibodies (1:5000 dilution) for 1 h at room temperature. Protein bands were visualized using an ECL chemiluminescent reagent kit (abs920, Absin, Shanghai, China) and imaged with a chemiluminescence detection system.

### 5.5. Immunofluorescence Staining

Human kidney organoids were fixed in 4% paraformaldehyde for 24 h, embedded in paraffin, and sectioned at 5 μm thickness using a microtome (Leica, Wetzlar, Hesse, Germany). Following deparaffinization and rehydration, sections were permeabilized with 0.5% Triton X-100 for 15 min and subjected to antigen retrieval by boiling in sodium citrate buffer (pH 6.0) for 15 min. Non-specific binding was blocked with 10% fetal bovine serum (FBS) in phosphate-buffered saline (PBS) for 2 h at room temperature. Sections were then incubated overnight at 4 °C with primary antibodies against NOS1 (sc-5302, Santa Cruz, Dallas, TX, USA; 1:100) and CCN1 (A1111, ABclonal; 1:100). After washing with PBS, sections were incubated with Alexa Fluor-conjugated secondary antibodies (1:500) for 1 h at room temperature. Nuclei were counterstained with DAPI, and slides were mounted with anti-fade mounting medium. Images were acquired using a confocal microscope (specify model if applicable).

### 5.6. Histological Analysis

Paraffin-embedded human kidney organoid sections (5 μm) were deparaffinized and stained with hematoxylin and eosin (H&E) using standard protocols. Briefly, sections were stained with hematoxylin for 5 min, differentiated in 1% acid alcohol, and counterstained with eosin for 2 min. After dehydration and clearing, slides were mounted with neutral balsam and imaged under a light microscope.

### 5.7. Reabsorption Assay

Organoids were incubated with 20 mg/mL 10 kDa dextran-rhodamine in culture medium for 6 h at 37 °C. Following incubation, organoids were washed five times with PBS to remove unincorporated dextran, fixed in 4% paraformaldehyde, and processed for paraffin embedding and sectioning (5 μm). Fluorescence signals were quantified using ImageJ.JS software.

### 5.8. ATP Quantification

Intracellular ATP levels in organoids were measured using the CellTiter-Glo 3D Cell Viability Assay (G9681, Promega, Madison, WI, USA) according to the manufacturer’s protocol. Briefly, culture medium was removed, and organoids were lysed in 100 μL of pre-warmed CellTiter-Glo 3D reagent. After 30 min of incubation on an orbital shaker at room temperature, luminescence was measured using a Synergy H1 plate reader (BioTek, Winooski, VT, USA).

### 5.9. Culture of Mouse Renal Tubular Epithelial Cells

Primary mouse renal tubular epithelial cells (RTECs) were isolated and cultured according to established methods [40]. Briefly, mice were anesthetized with 3% isoflurane and euthanized by cervical dislocation. Kidneys were immediately harvested and placed in ice-cold sterile PBS. Following removal of the renal capsule, the renal cortex was carefully dissected and minced into approximately 1 mm^3^ fragments using sterile surgical blades. The minced tissue was digested in 2 mg/mL collagenase type II (Worthington Biochemical, Lakewood, NJ, USA) at 37 °C for 60 min with gentle agitation. The digested suspension was filtered through a 40 μm cell strainer (Biologix, Jinan, Shandong, China) to obtain a single-cell suspension, followed by centrifugation at 300× *g* for 7 min at 4 °C. The cell pellet was washed once with 10 mL PBS and centrifuged again under identical conditions. Cells were resuspended in Kidney Culture Medium consisting of DMEM/F-12 (Gibco, Grand Island, NY, USA) supplemented with insulin (5 μg/mL), transferrin (2.75 μg/mL), selenium (3.35 ng/mL) (ITS; Invitrogen, Carlsbad, CA, USA), hydrocortisone (40 ng/mL; Sigma-Aldrich), recombinant human epidermal growth factor (10 ng/mL; R&D Systems, Minneapolis, MN, USA), and 1% antibiotic-antimycotic solution (10,000 U/mL penicillin, 100 μg/mL streptomycin, 0.25 μg/mL amphotericin B; Sigma-Aldrich). The cells were plated at a density of 1 × 10^5^ cells/cm^2^ and maintained at 37 °C in a humidified 5% CO_2_ incubator. The medium was replaced after 72 h and subsequently every 48 h until cells reached confluence.

### 5.10. Transient Transfection

RTECs were initially plated into 6-well plates and allowed to adhere for 12 h before undergoing transient transfection with 100 nM siRNA. The transfection was performed using Lipofectamine™ RNAiMAX (Invitrogen, Carlsbad, CA, USA) in accordance with the manufacturer’s protocols, and 36 h transfection duration was employed across experiments. The siRNA sequences utilized are detailed in Table 2.

### 5.11. 5-Ethynyl-2′-Deoxyuridine (EdU) Assay

Cell proliferation was assessed using the BeyoClick™ EdU Cell Proliferation Kit with Alexa Fluor 594 (C0078S, Beyotime) following the manufacturer’s protocol. Cells were incubated with 10 μM EdU in complete medium for 4 h at 37 °C. Following PBS washing, cells were fixed with 4% paraformaldehyde for 15 min and permeabilized with 0.3% Triton X-100 for 10 min. The Click reaction was performed for 30 min in the dark, followed by nuclear counterstaining with Hoechst 33342 (5 μg/mL) for 10 min. Fluorescent images were acquired using an inverted microscope (Nikon TS2) with consistent exposure settings across all samples.

### 5.12. Statistical Analysis

All data were analyzed using GraphPad Prism 10.0 (GraphPad Software, Boston, MA, USA). Normality was assessed using the Shapiro–Wilk test. For comparisons between two groups, an unpaired two-tailed Student’s *t*-test was applied for normally distributed data, while the Mann–Whitney U test was used for non-normally distributed data. Continuous variables are presented as mean ± standard deviation (SD). Statistical significance was defined as *p* < 0.05, with asterisks denoting significance levels: * *p* < 0.05, ** *p* < 0.01, and *** *p* < 0.001. All experiments included at least three biological replicates, with technical replicates performed in triplicate for each condition.

## Figures and Tables

**Table 1:** qRT-PCR Primer Sequences.

Gene	Forward Primer	Reverse Primer
*KIM-1*	TGGCAGATTCTGTAGCTGGTT	AGAGAACATGAGCCTCTATTCCA
*NGAL*	ATCACTCTCAGGGTCTGCAC	GGCAGGGGAATGTGAGAACT
*IL-18*	TTGTTGCGAGAGGAAGCGAT	GGAAAGAGCCTGTTTGAAGGC
*CCN1*	CTCGCCTTAGTCGTCACCC	CGCCGAAGTTGCATTCCAG
*PAPPA2*	AGAATAAGCCTGGCGATTTTGG	GGCCTTAGGTAGTTCCCAGC
*GDF15*	GACCCTCAGAGTTGCACTCC	GCCTGGTTAGCAGGTCCTC
*SEMA3F*	AACACAACCGACTACCGAATC	GGCTGCCCAGTGTATAATGAG

**Table 2:** siRNA Sequences.

	Sense (5′ → 3′)	Antisense (5′ → 3′)
siNC	UUCUCCGAACGUGUCACGUTT	ACGUGACACGUUCGGAGAATT
siCcn1 1#	CCCAGAACCAGUCAGAUUUTT	AAAUCUGACUGGUUCUGGGTT
siCcn1 2#	CCAGUGCACAUGUAUUGAUTT	AUCAAUACAUGUGCACUGGTT

## Data Availability

The original contributions presented in this study are included in the article and Supplementary Materials. Further inquiries can be directed to the corresponding authors.

## Supplementary Materials

The following supporting information can be downloaded at: https://www.mdpi.com/article/10.3390/toxins17090470/s1, Figure S1: Comprehensive analysis of gene expression changes following Stx2 treatment and associated cellular observations. (A) Volcano plot of differential gene expression 72 h post-Stx2 administration. A total of 3577 genes were significantly altered (1901 upregulated in red, 1676 downregulated in blue) compared to controls. (B) GO enrichment Analysis of Differentially Expressed Genes (DEGs) following Stx2 treatment. (C) KEGG pathway enrichment analysis of DEGs, highlighting key biological pathways affected by Stx2 treatment. (D) RT-qPCR analysis of DEGs in Mouse Kidney. *** *p* < 0.001. (E) Microscopic images of human kidney organoids. Scale bar, 50 μm. (F) Microscopic images of RTECs. Scale bar, 20 μm. (G) Immunofluorescence staining for epithelial cell marker EPCAM (red) in cultured RTECs. Scale bar, 10 μm.

## Abbreviations

The following abbreviations are used in this manuscript:

Stx	Shiga toxin
AKI	Acute kidney injury
STEC	Shiga toxin-producing *Escherichia coli*
HUS	Hemolytic uremic syndrome
Gb3	Globotrihexosylceramide
RTEC	Renal tubular epithelial cell
MD	Macula densa
RAAS	Renin–angiotensin–aldosterone system
PGE2	Prostaglandin E2
hiPSCs	Human induced pluripotent stem cells
JGA	Juxtaglomerular apparatus
