# Supplementary material for: Macula Densa Alleviates Shiga Toxin-Induced Acute Kidney Injury via CCN1-Mediated Renal Tubular Repair
Source: Toxins (Basel). 2025 Sep 21;17(9):470. doi: 10.3390/toxins17090470 (PMC12474279; doi:10.3390/toxins17090470)
Supplement: Supplementary file 1 [file toxins-17-00470-s001.zip › toxins-3827560-supplementary.pdf]

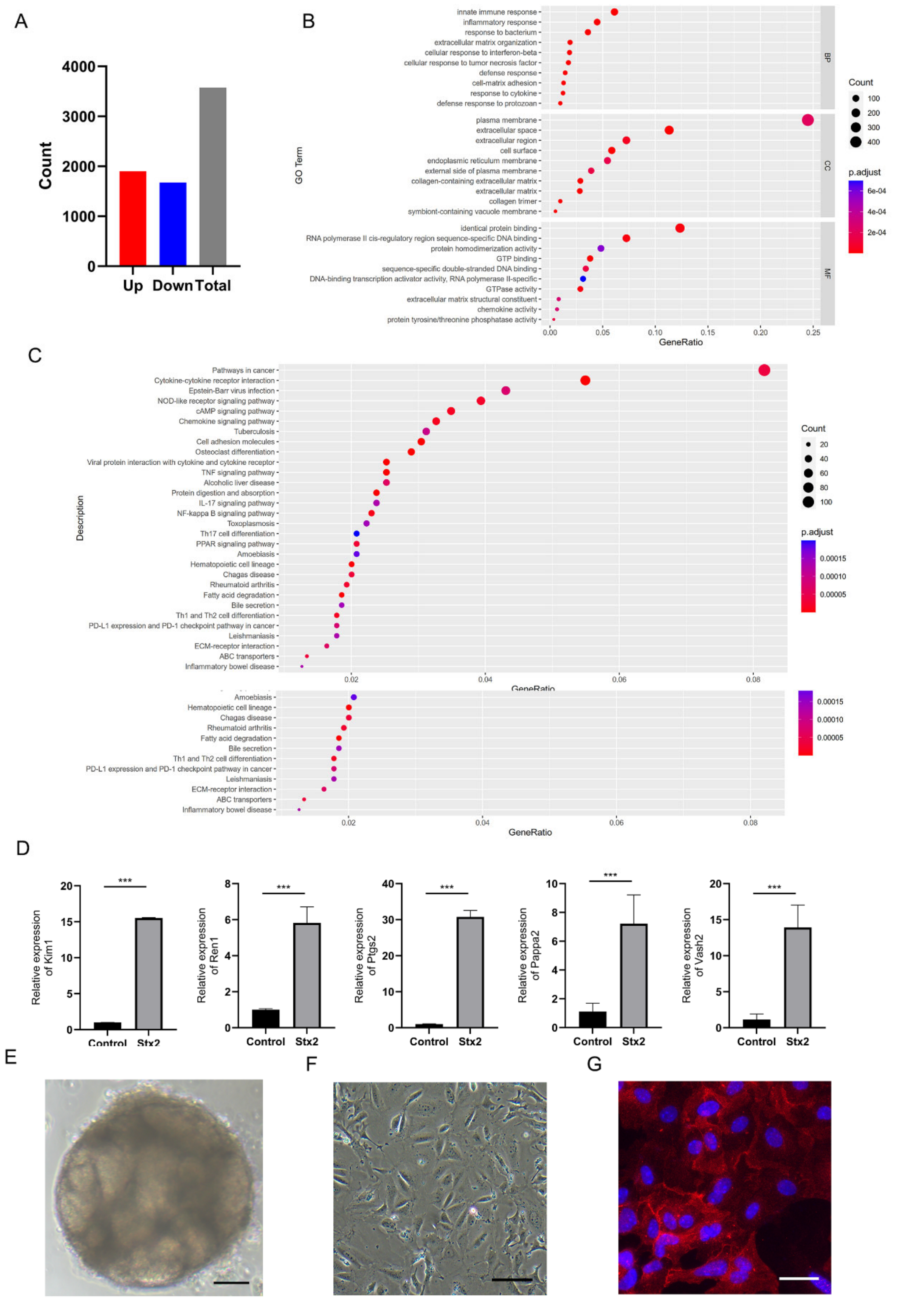

Figure S1. Comprehensive analysis of gene expression changes following Stx2 treatment and associated cellular observations. (A) Volcano plot of differential gene expression 72 hours post-Stx2 administration. A total of 3577 genes were significantly altered (1901 upregulated in red, 1676 downregulated in blue) compared to controls. (B) GO enrichment analysis of Differentially Expressed Genes (DEGs) following Stx2 treatment. (C) KEGG pathway enrichment analysis of DEGs, highlighting key biological pathways

affected by Stx2 treatment. (D) RT-qPCR analysis of DEGs in Mouse Kidney. ***$p < 0.001$.(E) Microscopic images of human kidney organoids. Scale bar, 50 μm. (F) Microscopic images of RTECs. Scale bar, 20 μm. (G) Immunofluorescence staining for epithelial cell marker EPCAM (red) in cultured RTECs. Scale bar, 10 μm.
